# Optimising Access to Care for Patients with Heart and Kidney Diseases: A World Heart Federation and International Society of Nephrology White Paper

**DOI:** 10.5334/gh.1460

**Published:** 2025-10-07

**Authors:** Jagat Narula, Javed Butler, Yazied Chothia, Debasish Bannerjee, Faical Jarraya, Ifeoma Ulasi, Valerie Luyckx

**Affiliations:** 1 University of Texas Health Houston, US; 2 Baylor Scott & White Health, Dallas, US; 3 Division of Nephrology, Department of Medicine, Faculty of Medicine and Health Sciences, Stellenbosch University & Tygerberg Hospital, ZA; 4 St George’s University of London, UK; 5 Nephrology, AGDUC, Montélimar, France; 6 Nephrology, CHU Hédi Chaker, Sfax, TN; 7 University of Sfax Faculty of Medicine, Sfax, TN; 8 College of Medicine, University of Nigeria, Enugu, NG; 9 Advocacy Working Group, International Society of Nephrology, US; 10 University Children’s Hospital Zurich, Switzerland; 11 Department of Public and Global Health, Epidemiology, Biostatistics and Prevention Institute, University of Zurich, CH; 12 Renal Division, Brigham and Women’s Hospital, Harvard Medical School, Boston, MA, US; 13 Department of Paediatrics and Child Health, University of Cape Town, ZA; 14 Chair, Advocacy Working Group, International Society of Nephrology, US

**Keywords:** cardiovascular disease, Chronic kidney disease, Public health

## Abstract

The clinical impact of diabetes medications including sodium-glucose cotransporter-2 (SGLT2) inhibitors, non-steroidal mineralocorticoid receptor antagonists (MRAs) and glucagon-like peptide 1 receptor agonists (GLP-1 RAs) on cardiovascular (CV) and kidney disease outcomes has focused attention on the inter-relatedness of kidney and heart health, both within and outside the context of diabetes. These conditions often co-exist in one individual resulting in frequent hospitalisations and premature deaths. Herein, we provide an updated comprehensive state-of-the-art review, summarising the linkages between heart disease and kidney disease, the mechanisms connecting these conditions, common risk factors, management, implications for health systems, and the impact on patients, particularly in low-resource settings. As experts representing the World Heart Federation (WHF) and International Society of Nephrology (ISN), we highlight areas of opportunity and provide recommendations on improving access to care for the growing numbers of patients with heart and kidney diseases with a focus on low-income and middle-income countries (LMICs).

## Introduction

Cardiovascular diseases (CVDs) are the leading cause of disease burden in the world (1). Kidney disease is an important contributor to this burden, and many risk factors are shared between CVD and kidney disease including diabetes mellitus, hypertension, dyslipidaemia, and elevated body mass index (BMI). According to the Global Burden of Disease (GBD) study, prevalent cases of CVD nearly doubled from 271 million in 1990 to 523 million in 2019, while the number of CVD deaths rose from 12.1 million in 1990 to 18.6 million in 2019 (1). In the same period, the prevalence of chronic kidney disease (CKD) also doubled from 342 million in 1990 to 697 million in 2019, and deaths rose from 0.6 to 1.4 million. Nearly two-thirds (63%) of people living with CKD (2) and over 75% of people with CVD reside in LMICs (3).

**Figure 1:**
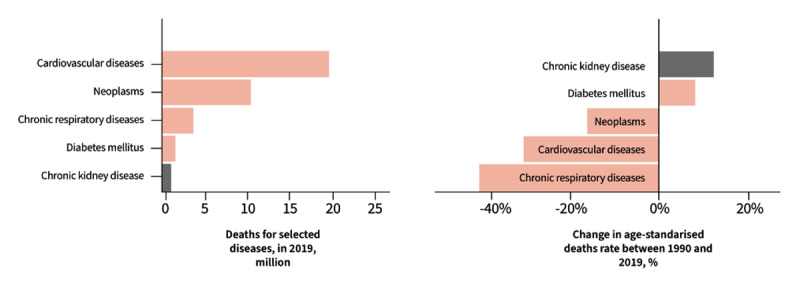
Comparison of global mortality and change in age-standardised death rates from selected noncommunicable diseases (12). Adapted from https://zenodo.org/records/8312881. Author: Boris Bikbov, Scientific-Tools. Org. Title: Comparison of global mortality from selected noncommunicable diseases. This figure is licensed under a Creative Commons Attribution NoDerivatives License: https://creativecommons.org/licenses/by-nc-nd/4.0/

In 2019, 3.1 million deaths were attributable to kidney disease (defined as estimated glomerular filtration rate [eGFR] less than 60 ml/min/1.73 m^2^ or urinary albumin to creatinine ratio [UACR] greater than or equal to 30 mg/g) (4). Of these deaths, 1.4 million were due to CKD itself and 1.7 million were cardiac deaths attributable to kidney disease, making kidney disease the 7^th^ leading global risk factor for death. More people with kidney disease therefore die of CVD (ischaemic heart disease [IHD], and stroke) before reaching kidney failure (KF) than die of KF itself (5). These numbers are even more striking within the population with diabetic nephropathy, of whom 90% die of CVD before reaching kidney failure (6). Concerningly, although the global age-standardised mortality rate for CVD declined by 30.9% between 1990 and 2017, this has increased by 13.3% for CKD in the same period (Figure 1). These diverging statistics highlight the beneficial impact of the global focus on reducing the CVD burden, whereas kidney disease has been relatively overlooked (7). It has been estimated that, globally, around 850 million people are living with some form of kidney disease, including acute kidney injury (AKI), CKD, and end-stage kidney failure (KF) (8). In addition, it is likely that the global deaths from kidney disease are also significantly underestimated, given that kidney disease is silent and diagnosis requires laboratory testing which is not accessible to all (9). Estimates suggest that at least 2 million additional people die annually of KF without access to kidney replacement therapy (KRT) and a further 1.7 million die of AKI, meaning the global mortality from kidney disease is likely significantly higher than reported in the GBD study (10). If nothing changes, kidney disease is anticipated to become the 5^th^ leading global cause of years of life lost by 2040 (11).

Increasing evidence suggests that the CV, kidney, and metabolic diseases, including diabetes and elevated BMI, are inextricably intertwined. This has led to the emergence of the terms ‘cardiovascular-kidney-metabolic (CKM) syndrome’ and ‘cardio-metabolic-renal’ (CMR) disease. A scientific statement by the American Heart Association (AHA) defined CKM syndrome as ‘a health disorder attributable to connections among obesity, diabetes, CKD, and CVD, including heart failure (HF), atrial fibrillation (AF), coronary heart disease (CHD), stroke, and peripheral artery disease,’ adding that ‘CKM syndrome includes those at risk for CVD and those with existing CVD’ (13). Diabetes, hypertension, hyperlipidemia, and obesity have been recognized as risk factors and co-morbidities of CVD for decades. The relationship between CVD and CKD has long been recognized by nephrologists, but its importance has only recently been acknowledged in the broader health care community. Thus, the focus here on the inextricable links between kidney disease and heart disease.

Given these trends and the increasing recognition of the interconnection between cardiovascular and kidney diseases, this white paper was developed to help summarise the current evidence, highlight shared challenges, and identify opportunities to improve access to care—particularly in low-resource settings. By bringing greater attention to the overlap between these conditions, the paper aims to support more integrated approaches in clinical practice and health policy.

## Methodology

This white paper was developed through a collaborative, multi-phase, process led jointly by the WHF and the ISN. The objective was to identify key challenges and opportunities for improving access to care for people living with CV and kidney diseases, particularly in LMICs.

The expert writing group comprising global experts in cardiology, nephrology, and public health was convened to guide the development of the paper. The group provided direction on the scope and structure of the content and offered feedback throughout the drafting process.

A targeted review of the literature was conducted to incorporate the most relevant global evidence, drawing on epidemiological data, clinical guidelines, health systems research, and implementation studies. Findings from the Global Burden of Disease (GBD) study and other key sources were used to inform the analysis. Real-world case examples were also included to illustrate practical approaches to integrated care and policy implementation across different settings.

While the white paper draws on the best available global data, we recognise that many of the studies and guidelines referenced originate in high-income countries, reflecting a wider gap in research investment and data generation in LMICs. This reinforces the urgent need to prioritise context-specific evidence from low-resource settings

Drafts of the white paper were reviewed by members of the expert writing group and other invited experts to ensure the content was evidence-based, contextually relevant, and actionable. Final revisions were made following this expert validation process.

### Connections between kidney disease and cardiovascular diseases

In a meta-analysis involving individual patient-level data on eGFR and urinary albumin-to-creatinine ratio from up to 27,503,140 individuals from 114 global cohorts, decreasing kidney function and increasing albuminuria were associated with myocardial infarction, HF, AF, stroke, peripheral vascular disease, and CV mortality (14). In addition to this meta-analysis, many individual studies have shown similar strong associations as detailed below. The connections between specific entities of CVD and kidney disease are therefore wide-ranging (Table 1).

**Table 1:** Heart – Kidney connections.


KIDNEY	CARDIAC CONSEQUENCES/ASSOCIATIONS

Albuminuria/proteinuria	Major risk factor for cardiovascular disease (CVD) (15) and cardiovascular (CV) mortality (16)

Chronic kidney disease (CKD)	More deaths from CVD than kidney failure Major risk factor for CVD 17–50% have heart failure (6,17,18,19)

Acute kidney injury (AKI)	Due to heart failure Cause of heart failure (6,18,19,20,21,22)

Worsening renal function	Due to heart failure Cause of heart failure (17)

Kidney transplant (23)	Heart diseases are the leading cause of death Heart disease can make candidates ineligible for a transplant or delay transplantation Challenges with cardiac medication dosing and interaction with immunosuppressants

Haemodialysis (17,23)	Heart disease is a major cause of death Sudden cardiac death Atrial fibrillation Heart failure (17) Ischaemic heart disease Valvular heart disease Challenges with therapy/medication dosing and approval to use

Vascular access for dialysis	Haemodialysis catheters are a risk for infective endocarditis Arteriovenous Fistulas may contribute to heart failure (23)

**HEART**	**KIDNEY CONSEQUENCES**

Ischaemic heart disease	Contrast-induced acute kidney injury (AKI) AKI post bypass surgery Atheroembolic kidney disease (19,24,25)

Heart failure	49% have kidney disease (albuminuria or reduced glomerular filtration rate [GFR]) (17,19,25)

Atrial fibrillation	Therapeutic challenges especially in haemodialysis (26)

Aortic stenosis	Progression on dialysis (17)

Rheumatic fever	AKI, post-infectious glomerulonephritis (20,21)

Peripartum cardiomyopathy	AKI

Cardiac surgery	AKI

Heart transplant	High risk of CKD, KF (heart function, side effect of immunosuppression)

Infective endocarditis	Immune complex glomerulonephritis Ischaemic acute tubular necrosis (ATN) (sepsis, CRS) Nephrotoxic ATN (aminoglycosides) Acute tubulointerstitial nephritis (beta-lactam antibiotics) Renal infarcts Renal abscesses secondary septic emboli

**SYSTEMIC DISEASES IMPACTING KIDNEY AND HEART**	

Diabetes mellitus (DM)	1 in 3 have CKD Major risk factor for CVD Overlap DM, CKD, CVD (cardio-kidney-metabolic syndrome [CKM] in around 1:20 people in US (CKM) 9 in10 people with Diabetes and CKD dies of heart disease before developing kidney failure (6,27)

Hypertension	1 in 5 have CKD Major cause of heart failure, CVD (27)

Overweight/obesity	Risk factor for worsening CKD, heart disease (27)

Sepsis	Important cause of kidney and heart failure (20)

Autoimmune and other related diseases: SLE, vasculitis, sarcoidosis	Major cause of glomerulonephritis, can cause pericarditis, cardiomyopathy, arrhythmias, conduction abnormalities

Cancers (28)	Drugs may be toxic to kidney and heart Tumour lysis syndrome causing AKI and cardiac arrhythmias secondary to hyperkalaemia

Preeclampsia	Short- and long-term risks of kidney and heart disease (29)

Low birth weight, preterm birth	Long-term risk of hypertension, CKD, heart disease, DM (30,31)

Genetic conditions	Fabry disease, amyloidosis (32)

**SOCIAL DETERMINANTS OF HEALTH (10,30,33)**	

Poverty	CKD, AKI, rheumatic fever, heart failure, IHD

Nutrition	CKD, AKI, IHD, heart failure, MIA syndrome

Education	CKD, AKI, IHD, heart failure

Race, ethnicity	CKD, heart failure

Sex	CKD, AKI, peripartum cardiomyopathy

Geography	CKD, AKI, heart failure, IHD

Climate change	CKD, AKI, kidney failure, heart failure, IHD

Lifestyle	CKD, heart failure, IHD


AKI: acute kidney injury; ATN: acute tubular necrosis; CKD: chronic kidney disease; CKM: cardiovascular-kidney-metabolic; CRS: cardiorenal syndrome; CV: cardiovascular; CVD: cardiovascular disease; DM: diabetes mellitus; GFR: glomerular filtration rate; IHD: ischaemic heart disease; KF: kidney failure; MIA: malnutrition-inflammatory-atherosclerosis; SLE: systemic lupus erythematosus.

In light of these patterns and the growing recognition of how cardiovascular and kidney diseases intersect, this white paper was developed to consolidate current knowledge, elevate awareness of shared risk factors, and highlight areas where coordinated action could improve patient outcomes. The goal is to support more integrated and equitable approaches to care, particularly in settings where health system capacity is limited and the burden of disease is high.

### Heart failure and left ventricular hypertrophy

The Atherosclerosis Risk in Communities (ARIC) study in 14,857 middle-aged adults demonstrated that those with moderately/severely reduced kidney function (as defined by eGFR) were at high risk for developing HF (34). The incidence of HF was three-fold higher in those with eGFR <60 ml/min/1.73 m^2^ compared to the reference group with eGFR >90 ml/min/1.73 m^2^ (18 vs. 6 per 1000 person-years). The overall adjusted relative hazard of developing HF was 1.94 for individuals with eGFR <60 ml/min/1.73 m^2^ compared to the reference group.

**Figure 2:**
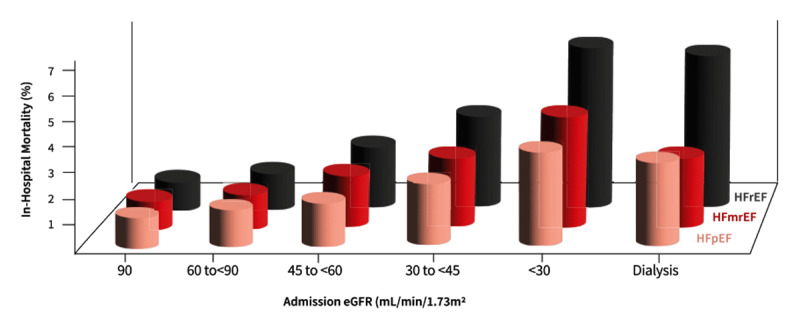
Mortality increases with heart failure and worsening kidney function (37). Adapted from the Journal of the American College of Cardiology, volume 78, issue 4, author: Patel RB et al., title: Kidney function and outcomes in patients hospitalized with heart failure, pages 330–343, Copyright 2021, with permission from Elsevier.

Decreasing eGFR and increasing proteinuria both increased the chance of HF in a community study of 3,971 patients in the Chronic Renal Insufficiency Cohort (35). The presence of CKD in HF, which occurs in approximately half of all HF patients, is associated with increased mortality and hospitalisation (Figure 2) (36).

In a study of 365,494 patients hospitalised with HF, 234,332 (64%) had eGFR <60 ml/min/1.73 m^2^ and 18,869 (5%) were on dialysis (37). Mortality increased significantly in a graded fashion with progressively declining admission eGFR (37). Steep associations were found between admission eGFR and across ejection fraction subgroups but were stronger for HF with reduced ejection fraction (HFrEF) compared with HF with mid-range (HFmrEF) or preserved ejection fraction (HFpEF) (p interaction = 0.045) (37).

In a cohort of maintenance haemodialysis patients, seven in ten had HF, of which 81% was HFpEF and 19% was HFrEF (38). Patients with HFrEF had a greater than three-fold higher risk of CV hospitalisation and/or cardiac death compared to those without HF, with an adjusted hazard ratio (HR) of 3.24 (95% confidence interval [CI] 1.08, 9.75). The association with HFpEF did not reach statistical significance (38). The prevalence of left ventricular hypertrophy (LVH) increases in a stepwise fashion as kidney function deteriorates and 70–80% of patients with KF present with established LVH (39). In a study of African Black patients with chronic kidney failure, 95% had LVH and 85% had hypertension at first presentation (40).

### Coronary heart disease

In a study of 14,971 individuals, reduced eGFR was associated with a higher risk of recurrent CHD events and mortality from CHD and all causes (41). The associations were significantly greater among those with anaemia. The adjusted relative hazards of all-cause mortality associated with moderately decreased versus normal kidney function (GFR 30–59 vs. ≥90 ml/min per 1.73 m^2^) were 1.7 (95% CI 1.3, 2.2) without and 3.5 (95% CI 2.4, 5.1) with anaemia (p interaction = 0.001).

Albuminuria *per se*, even in the setting of a normal eGFR is associated with an increased risk of IHD, which is further markedly increased in the setting of a reduced eGFR (42,43). Indeed, the ESC 2021 guideline on CVD prevention has included albuminuria in their ABCDE screening recommendations (albuminuria, blood pressure, cholesterol, diabetes, eGFR) (44). It has also been shown that the relative risk of CV mortality increases as eGFR declines and UACR rises (18).

### Acute coronary syndrome

CKD impacts outcomes in patients with acute coronary syndromes (ACS). Among 2,706 patients with acute myocardial infarction (AMI) or unstable angina, kidney disease was associated with a higher odds of death, with odds ratios (ORs) of 1.76 (95% CI 0.93, 3.33), 2.72 (95% CI 1.43, 5.15), and 6.18 (95% CI 3.09, 12.36) for mild (eGFR 60–89 mL/min/1.73 m^2^), moderate (eGFR 30–59 mL/min/1.73 m^2^), and severe (eGFR <30 mL/min/1.73 m^2^) kidney disease, respectively, compared to those with normal eGFR (45). The associations between kidney disease and mortality rate were similar in the AMI and unstable angina subgroups (45).

In a population based study from Alberta, Canada, the unadjusted rates of AMI were highest among those with prior myocardial infarction (18.5 per 1000 person years), followed by CKD (eGFR <60 ml/min/1.73 m^2^) with diabetes (12.9 per 1000 person years), CKD alone (6.9 per 1000 person years) and diabetes alone (5.4 per 1000 person years), although the associations with CKD were attenuated after adjustment for demographic and clinical factors (43). Mortality after AMI was however highest among those with CKD and diabetes, followed by CKD alone, prior AMI and diabetes alone.

### Sudden death

Sudden cardiac death (SCD) is the most common cause of death in people on dialysis, and is responsible for nearly one in four deaths in this group (46). Bradyarrhythmias have been implicated as a potential cause (46). It has been postulated that the high prevalence of LVH in patients with CKD, along with the accompanying risk of cardiac rhythm disturbances, may in part explain the elevated likelihood of SCD in CKD (47,48). Abnormal electrolyte levels could also contribute to the excess SCD risk especially in those on dialysis (48).

### Valvular heart disease

CKD is a major risk factor for valvular heart disease (VHD) (49). VHD in general, and particularly mitral regurgitation and aortic stenosis, is associated with significantly reduced survival in patients with CKD (49). Approximately 2–6% of patients on chronic haemodialysis develop infective endocarditis (IE), an incidence that is 50–60 times higher than in the general population (50). In a study of patients who underwent surgery for IE, mortality rates rose with the severity of underlying CKD (51). Mortality rates at 30 days and 1 year were 5.6% and 15.5%, respectively, in patients without CKD, rising to 38.1% and 75.6%, respectively, in those on haemodialysis (51). In a study of patients who underwent surgery for IE, mortality rates rose with the severity of underlying CKD (51). Mortality rates at 30 days and 1 year were 5.6% and 15.5%, respectively, in patients without CKD, rising to 38.1% and 75.6%, respectively, in those on haemodialysis (51).

### Atrial fibrillation

Also from ARIC (n = 10,328), compared to individuals with normal GFR (cystatin C-based GFR [eGFR_cys]_ ≥90 mL/min/1.73 m^2^), the HRs for AF in individuals with mildly decreased kidney function (eGFR_cys_ of 60–89 mL/min/1.73 m^2^), CKD stage 3 (30–59 mL/min/1.73 m^2^), and CKD stage 4 (15–29 mL/min/1.73 m^2^) were 1.3 (95% CI 1.1,1.6), 1.6 (95% CI 1.3, 2.1), and 3.2 (95%CI 2.0, 5.0), respectively (p for trend <0.0001) (52). Moderate albuminuria, previously called microalbuminuria (UACR 30–299 mg/g), and macroalbuminuria (UACR ≥300 mg/g) were also associated with 2.0 and 3.2 higher hazards of AF, respectively, compared to those with normal UACR levels (<30 mg/g). Participants with both CKD stage 4 and high levels of albuminuria had a particularly high AF risk, with a HR of 13.1 (95% CI 6.0, 28.6) compared to those with normal UACR levels and normal GFR (52). More recent studies indicate that AF and CKD coexist and share the same risk factors, but the direction of association is unclear (53). Using bidirectional summary-level Mendelian randomization analysis on a genome-wide association study for eGFR, involving 765,348 European individuals with CKD and 588,190 with AF, a genetic predisposition to AF was significantly associated with a reduced eGFR [for log-eGFR, beta –0.003 (standard error, 0.0005), p < 0.001] and an increased risk of CKD [beta 0.059 (0.0126), p < 0.001] (54). The reverse; however, was not observed, in that a genetic predisposition to reduced eGFR was not associated with increase AF.

### Stroke

In the ARIC cohort (n = 13,716), CKD was associated with a nearly two-fold increased risk of stroke after adjustment for known risk factors (HR 1.81; 95% CI 1.26, 2.02), an effect that was particularly pronounced in the presence of anaemia (HR 5.43; 95% CI 2.04, 14.41) (55). Kidney disease, determined by eGFR, was related to incident stroke in an inverse step-wise fashion, with risk increasing by 3-, 4.1-, 5.4-, and 7.1-fold for CKD stage 3 to 5 and dialysis patients compared with the general population (56). The REasons for Geographic and Racial Differences in Stroke (REGARDS) Study, a two-year follow-up of 20,386 participants, documented an incidence of stroke symptoms of 20.7% with an eGFR <45 ml/min per 1.73 m^2^ (HR: 1.26) and 18.8% with an ACR >300 mg/g (HR 1.29, p = 0.005 for trend) (57). Also, in a 2015 meta-analysis of 63 cohort and 20 randomised controlled studies, stroke risk increased by 7% for every 10 ml/min/1.73 m^2^ decline in eGFR and 10% per 25 mg/mol increase in ACR independent of GFR. (58)

### Peripheral arterial disease

Further data from ARIC (n = 14,280) uncovered an increased risk for incident peripheral arterial disease in participants with CKD, with a multivariable adjusted relative risk of 1.56 after adjustment for CVD risk factors (95% CI 1.13, 2.14) (59). In a large meta-analysis of the CKD-Prognosis Consortium report which included more than 800,000 subjects, the risk for developing peripheral arterial disease increased by 22% when eGFR decreased from a baseline of 95 ml/min/1.73 m^2^ to 45 ml/min/1.73 m^2^ and by 2-fold for eGFR of 15 ml/min/1.73 m^2^ (60).

### Demographics of kidney disease

CKD is common. Pooled results of 33 population-based representative studies in 2010 revealed an age-standardised prevalence of CKD stages 1–5 in individuals aged >20 years of 10.4% among men and 11.8% among women. CKD age-standardised prevalence was lower in high-income countries: 8.6% and 9.6% in men and women, respectively compared to 10.6% and 15.5% in low and middle-income countries (61). A more recent comprehensive systematic review and meta-analysis of 100 studies involving almost 7 million participants noted a global prevalence of 13.4% for CKD stages 1–5 and 10.6% for CKD stages 3–5) (62). Similar commonality and patterns are observed for CVD, with prevalence nearly doubling from an estimated 271 million people in 1990 to 523 million by 2019 (63). Lower-middle and low-income countries had the highest age-standardized CVD prevalence rates in 2019, at 7.4% compared to 6.9% across high-income countries. Variations across cardiovascular disease subtypes exist however, with ischemic heart disease and stroke more prevalent in low- and middle-income countries, and atrial fibrillation more prevalent in high-income countries (63,64).

A study of 11,607 US adults (mean age 48.5 years, 51.0% women) found that participants with a high cardiac, renal, and metabolic comorbidity burden were more likely to be older, male, of non-Hispanic Black race or ethnicity, unemployed, of low socioeconomic status, and without a high school degree (65).

A review of US CKD patients highlighted that the likelihood of KF was nearly four-fold higher in Blacks compared with White individuals (18), with diabetes and hypertension being the primary causes of KF among Blacks. Hispanics were nearly 1.3 times more likely to be diagnosed with KF compared to non-Hispanic individuals. Racial and ethnic minorities, including African Americans in the US, Indigenous groups in Canada and Australia, and Indo-Asians in the UK, were disproportionately affected by advanced and progressive kidney disease (66).

Sex differences have also been reported. Globally, the prevalence of CKD is higher in women but men progress more rapidly to KF (67). Global CVD prevalence and mortality rates are higher in men, however important sex differences in CV phenotypes, risk factors and care outcomes are observed for different cardiovascular disease subtypes, including a potentially higher risk of developing CKD due to hypertension in women than men (68). Female CKD patients have lower risks of CV events, CV mortality and mortality from any cause than their male counterparts—differences which could not be explained by risk factors (69). Therefore, sex impacts the interaction between CKD and CVD, although the pathophysiology is not yet well understood.

### Children

Links between heart and kidney disorders have also been found in children. The Cardiovascular Comorbidity in Children with CKD Study of 6–17 year-olds with an initial eGFR of 10–60 mL/min/1.73 m^2^ found that 26.1% had uncontrolled hypertension (n = 545), with the prevalence increasing from 24.4% in CKD stage 3 to 47.4% in CKD stage 5 (70). LVH was also more prevalent with increasing severity of CKD, from 10.6% in CKD stage 3a to 48% in CKD stage 5 (70). A study of 1,410 children with congenital heart defects found that 7.4% had congenital anomalies of the kidney and urinary tract (CAKUT) (71). There were no differences in the prevalence of CAKUT according to maternal age, sex, parity, gestational age or history of medication during pregnancy (71). The reported prevalence was substantially higher than the 0.5% observed in the general population (72). A study in mouse models found that 29% of mutations causing congenital heart disease also caused kidney anomalies (72).

### Pregnancy, the kidney, and cardiovascular disease

Hypertensive disorders of pregnancy are common, affecting up to 15% of women during their reproductive lives (73). These disorders are more common in lower resource settings and have important short- and long-term impacts on maternal and foetal kidney and CV health (74). Kidney disease is a major risk factor for hypertensive disorders of pregnancy. A meta-analysis of 23 studies including 5,769,891 pregnant women found that preeclampsia was associated with an increased risk of CKD (pooled adjusted risk ratio [aRR] 2.11), KF (aRR 4.90), and kidney-related hospitalisation (aRR 2.65). Gestational hypertension was associated with 1.49-fold and 3.64-fold raised risks of CKD and KF, respectively (75).

A Swedish national cohort study of 2.2 million women with a singleton delivery found that 11,572 (0.5%) were diagnosed with CKD during 56 million person-years of follow-up (76). Within 10 years following delivery, the HR for women developing CKD was 7.12 for other hypertensive disorders, 4.38 for preeclampsia, 3.50 for preterm delivery, 3.15 for gestational diabetes, and 1.22 for those with small for gestational age infants. Risk remained significantly elevated 30–46 years after delivery. Women with more than one adverse pregnancy outcome were at even greater risk.

A meta-analysis reported that a prevalence of AKI in women with hypertensive disorders of pregnancy (HDP) in Africa of 6%, with preeclampsia and eclampsia being the most common types of HDP (77). However, the authors suggested that this may be an underestimate because many studies did not use standard diagnostic criteria, and many studies were performed by obstetricians rather than nephrologists and earlier stages of AKI may have been missed (77).

A meta-analysis of 22 studies >6.4 million women, of whom >258,000 had preeclampsia, demonstrated that preeclampsia was independently associated with an increased risk of future HF (risk ratio [RR] 4.19; 95%CI 2.09, 8.38), CHD (RR 2.50; 95%CI 1.43, 4.37), CVD death (RR 2.21; 95% CI 1.83, 2.66), and stroke (RR 1.81; 95% CI 1.29, 2.55) (78).

### Mortality

Overall, 9% (1 in 10) of CVD deaths in 2019 are attributable to kidney disease—which causes 13% of deaths from IHD and 8% of deaths from stroke (79). CVD is responsible for up to 50% of the mortality risk in patients on dialysis, with the largest relative excess observed in younger patients (80). The risk of death due to CVD is 10–20 times greater in dialysis patients compared to age- and sex-matched controls in the general population (80). In the US in 1997, the National Kidney Foundation established a Task Force to investigate outcomes of CVD in the general population compared to those with CKD. Their findings revealed that the annual CV-related mortality rate among patients undergoing KRT stood at approximately 9%, a figure nearly 30 times higher than that observed in the general population. Of heightened concern was the fact that CV fatalities among KRT patients aged 25–34 were comparable to those seen in individuals in the general population aged over 85, irrespective of sex or ethnicity (81).

### Life course risks

**Figure 3:**
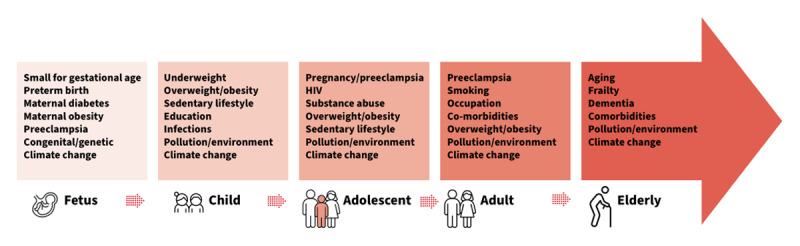
Examples of factors contributing to heart and kidney risk over the life course (88) Adapted from https://www.sciencedirect.com/science/article/pii/S2468024919315864. Authors: Valerie A. Luyckx, David Z.I. Cherney, Aminu K. Bello. Title: Preventing CKD in developed countries. This figure is licensed under a CC BY-NC-ND license (http://creativecommons.org/licenses/by-nc-nd/4.0/).

The risk of premature non-communicable diseases (NCDs) may begin during foetal life (Figure 3). This paradigm was proposed by David Barker who observed an increase in cardiovascular deaths among adults who had been of low birth weight (82). Around the same time Brenner and colleagues hypothesised that low birth weight and preterm birth are associated with a congenital reduction in nephron number, which would predispose to elevated blood pressure and a higher likelihood of kidney disease in later life (83,84). In population-based studies, preterm birth (gestational age <37 completed weeks) and low birth weight for gestational age have been associated with increased risks of CV and kidney disorders in later life (85,86). Over 35 million babies worldwide are born too small or too early, suggesting many children begin life at risk of heart and kidney disease (87). More specifically, low birth weight and prematurity are risk factors for hypertension, proteinuria, and CKD later in life (83,85). High birth weight, particularly when due to maternal diabetes, is also detrimental to the heart and kidneys, with increased risks of congenital heart disorders, and obesity/metabolic syndrome, proteinuria and kidney disease in later life (83).

Using data (CV health albuminuria [CVH], a composite of seven metrics) from the Coronary Artery Risk Development in Young Adults Study (CARDIA), investigators demonstrated that less-than-ideal CVH in young adults aged 18 to 30 predicted premature CV morbidity and mortality three or more decades later (89). Of concern, over two-thirds had moderate-poor CVH at baseline, more prevalent among Blacks compared with Whites, who then had twice (0.9 vs. 0.5/1,000 person-years) the rates of CVD during follow-up. Poor CVH established as covert subclinical disease in young adults progresses to overt CVD morbidity and mortality in mid-to late-life (90). Conversely, an ideal CVH at this age predicted a low absolute and relative risk of premature CVD in later life (89). The mechanisms involved in the evolution of poor CVH in early adulthood culminating in CVD and CKD risk over time and causing increased morbidity and mortality in late adult life, are multiple, including early life factors, social and structural factors, and health and lifestyle (91). Obesity is a major modifier of these early risks (92,93,94).

Developing a model with a multisectoral approach incorporating healthcare measures and socioeconomic and health policies that promote equity, access to quality care, information, education, and healthy life-style counselling starting in childhood and adolescence are ideal strategies to reduce CVD and CKD risk throughout the life course to reduce morbidity and mortality attributed to NCDs. This unique window is often not fully harnessed in many high-income countries (HICs) (95) or even explored in LMICs.

### Multimorbidity burden

Given the shared risk factors, CVD and CKD often occur together as well as with other disorders. Multimorbidity is especially common among people with kidney disease. In a population-based study adults in Alberta, Canada, patients seen by nephrologists had the highest mean number of comorbidities (4.2; 95% CI, 4.2–4.3 vs [lowest] 1.1; 95% CI, 1.0–1.1) and the highest mean number of prescribed medications (14.2; 95% CI, 14.2–14.3 vs [lowest] 4.9; 95% CI, 4.9–4.9) (96). The mean number of comorbidities in patients seen by cardiologists was 2.6 (95% CI, 2.6–2.6) with a mean number of prescribed medications of 8.7 (95% CI, 8.7–8.7).

In a study of 10,336 people from South Africa, the prevalence of multimorbidity, defined as having ≥2 health conditions, was 20.7% (95% CI 19.5, 21.9) and was higher in females (26.2%) compared to males (14.8%) (97). The most common conditions, measured using biomarkers, were hypertension (45%), anaemia (24.7%), HIV (19.6%), and diabetes (11.7%) (97). This study illustrates the high prevalence of these combined risk factors for CKD and CVD in the community. The REPORT-HF global cohort study of 18,553 patients from 358 hospitals in 44 countries on six continents found that in patients with acute HF, more than half (51%) of patients with ≥5 comorbidities (n = 4,449) had CKD (98). The highest prevalence of multimorbidity was observed in North America and Europe and the lowest in southeast Asia and the western Pacific. Fewer patients from LMICs had multimorbidity compared with patients in HICs (73% vs. 85%, p < 0.0001). The proportion of 1-year all-cause mortality risk in acute HF patients that could be explained by the presence of ≥5 comorbidities was greatest in HICs (61%), followed by LMICs (31%), and upper-middle-income countries [UMICs] (27%).

Among 11,607 US adults with a mean age of 48.5 ± 0.4 years, at least one cardiac, kidney or metabolic (CKM) condition was present in 26.3%, 8% had at least two conditions and 1.5% had at least three (65). CKD was the most prevalent CKM condition (13.9%), followed by diabetes (13.3%) and CVD (8.6%). The CKM burden was higher among non-Hispanic Black subjects and among those with lower socioeconomic status and higher unemployment.

## Pathophysiology of the Interrelationship Between CVD and Kidney Disease

The mechanisms connecting CKD and CVD are multifactorial and are intricately linked with hypertension, diabetes mellitus (DM) and body mass index (BMI). Hypertension is a strong risk factor for CVD in patients with CKD, including AMI, HF, arrhythmia, stroke, and peripheral vascular disease (24). Persistently high blood pressure can also worsen kidney dysfunction and, conversely, declining kidney function can result in deteriorating blood pressure control (24). A meta-analysis of 45 cohorts (25 general population, 7 high-risk, and 13 CKD cohorts), including 1,127,656 participants, of whom 364,344 had hypertension, demonstrated that both low eGFR and high albuminuria were associated with mortality in non-hypertensive and hypertensive individuals in the general population and in high-risk cohorts (99). Another meta-analysis in 1,024,977 participants (128,505 with DM) from 30 general population and high-risk CV cohorts and 13 CKD cohorts found that despite higher risks for mortality and KF in patients with DM, the relative risks of these outcomes by eGFR and UACR were much the same irrespective of the presence or absence of diabetes (100). It is increasingly being recognised that reduced kidney function and albuminuria are important independent CV risk factors as discussed above (43,99,100).

While the risks of all-cause and CV death increase exponentially with decreasing kidney function (24,101), it is well established that the shared traditional atherogenic risk factors in CVD and CKD—including hypertension, dyslipidaemia, DM, obesity and smoking—do not completely explain the elevated risk of CV mortality in CKD patients compared to the general population (80,102,103).

The classical CV risk factors appear to be implicated in the early stages of CKD, while in the intermediate and late stages, non-traditional risk factors such as sodium retention, volume expansion, anaemia, inflammation, malnutrition, sympathetic overactivity, mineral bone disorders, accumulation of uraemic toxins, and hormonal disorders accelerate CVD progression (18).

The combination of endothelial dysfunction, low-grade inflammation, and dyslipidaemia associated with both incipient and progressive kidney disease may be responsible for the acceleration of atherosclerosis and, accompanied by hypertension, could be responsible for the high prevalence of coronary ischaemia and CV events in patients with CKD (104). All-cause mortality increases sharply with the addition of each individual component of the so-called malnutrition, inflammation, and atherosclerosis (MIA) syndrome in patients with KF (105). Similarly, the pathophysiology of stroke in those with CKD has also been found to encompass both shared traditional risk factors including hypertension and DM and non-traditional CKD-related factors such as chronic inflammation, uraemic toxins, reactive oxygen species, anaemia, and CKD-mineral-bone disorder, potentially through triggering vascular injury and endothelial dysfunction (56).

**Figure 4:**
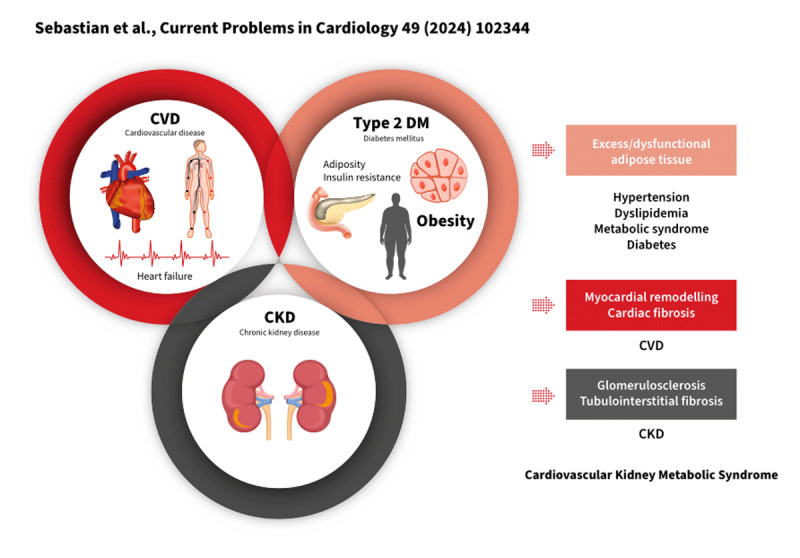
Cardiovascular-kidney-metabolic syndrome (106). Adapted from Current Problems in Cardiology, volume 49, issue 2, author: Sebastian SA et al., Title: Cardiovascular-kidney-metabolic (CKM) syndrome: A state-of-the-art review, page 102334, Copyright 2023, with permission from Elsevier.

The emergence of the term CKM syndrome recognises that metabolic abnormalities have a central pathophysiological role in the bidirectional interaction between the CV and kidney systems (Figure 4) (13). Molecular mechanisms contributing to this syndrome include hyperglycaemia, insulin resistance, elevated renin-angiotensin-aldosterone system (RAAS) activity, the generation of advanced glycation end-products, oxidative stress, lipotoxicity, endoplasmic reticulum stress, abnormal calcium handling, mitochondrial dysfunction with impaired energy production, and chronic inflammation (106). Meanwhile, the impact of CKM syndrome on vascular integrity, atherogenesis, myocardial function, haemostasis, and cardiac conduction results in higher risks of CVD such as CHD, stroke, HF, peripheral arterial disease, AF, and SCD (13).

### Obesity

Obesity is associated with both progression of kidney disease and CVD. Data from the ARIC study found a consistent association between midlife obesity status (measured by BMI, waist-to-hip ratio, predicted percent fat at baseline) and development of KF in White and Black women, but not in White men (107). Among patients with glomerulopathies and proteinuria, obesity is associated with adverse cardiovascular and renal outcomes, but the association with kidney disease progression attenuates with multivariate analysis (108,109). The association between obesity and multiple forms of CVD is strong (110). Weight loss is associated with reduction in risk factors for CVD and CKD; however, the risk of CVD has been found to be reduced after surgical but not medical weight loss (110). Bariatric surgery has also been associated with improvement in CKD risk (111). This may relate to the degree of weight loss achieved and is likely mediated by accompanying improvements in additional risk factors such as hypertension and blood glucose (110).

Epidemiological studies have shown that visceral adipose tissue (VAT) is associated with increased metabolic risk and overall mortality, whereas subcutaneous adipose tissue (SAT) ameliorates insulin sensitivity and reduces the risk of type 2 DM (112). These risks are affected by genetic, biological and lifestyle factors including physical activity, nutrition, and stress (112). An analysis of African-Americans enrolled in the Jackson Heart Study reported that abdominal VAT and SAT were both associated with adverse cardiometabolic risk factors, but VAT was more strongly associated with these risk factors (113). A study of 5,113 individuals (2,933 Inuit, 1,397 Africans, and 783 Europeans) from three studies in Greenland, Kenya, and Denmark indicated that ethnicity plays a role in the pathway from abdominal fat to selected cardiometabolic risk factors (114). Across ethnic groups and sex, a one standard deviation increase in VAT was associated with higher levels of hepatic insulin resistance (ranging from 14% to 28%), triglycerides (8% to 16%) and lower high-density lipoprotein cholesterol (–1.0 to –0.05 mmol/L) independent of BMI (114). However, VAT showed positive associations with many other cardiometabolic risk factors in Inuit and Europeans but not in Africans. SAT was mainly associated with the outcomes in Inuit and Africans (114).

The Framingham Offspring Study reported that VAT and SAT were associated with CKD when defined using cystatin C estimating equations but not when using a creatinine-based estimating equation (115).

In contrast, epidemiological studies have consistently reported reduced all-cause mortality in obese haemodialysis patients. This reverse epidemiology has been referred to as the obesity paradox among patients on hemodialysis. The prospective Dialysis Outcomes and Practice Patterns Study (DOPPS) that included 9,714 haemodialysis patients from the US and Europe reported a statistically significant inverse linear correlation between relative mortality risk and BMI (p < 0.0001). Relative mortality risk was significantly lower in both US (relative risk 0.7, p = 0.002) and European (relative risk 0.61, p = 0.01) haemodialysis patients who had a BMI ≥30 kg/m^2^ compared to those with a BMI of 23.0–24.9 kg/m^2^ (116). Postulated mechanisms for this survival advantage include improved haemodynamic stability, altered cytokine profiles, uraemic toxins sequestered with fat tissue, and altered endotoxin-lipoprotein response (117). An obesity paradox has also been described in CVD, potentially related to earlier diagnosis among individuals with class 1 obesity, better cardiorespiratory fitness and more physical reserve (110).

### Social determinants of health

**Figure 5:**
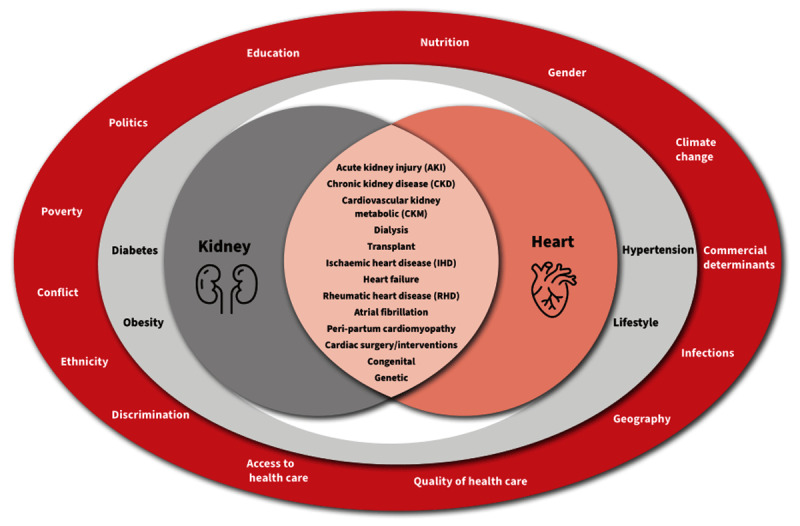
Social determinants of health and CKM.

The risk of NCDs, including CVD and CKD, starts in utero and is further impacted by epigenetic factors, lifestyle choices and socioeconomic circumstances throughout the life course. The United Nations (UN) Sustainable Development Goals (SDGs) emphasise wellbeing at all ages (118) and have been noted as an opportunity to stem the tide of NCDs at the source (119). CKM particularly affects populations with poor social determinants of health (SDH), which include the economic, social, environmental, and psychosocial factors that influence health (Figure 5) (65,120,121). The SDH that influence CV health have been subdivided into sociopolitical and economic context, social and community context, and lived personal experience (120). The sociopolitical and economic context includes economic stability, education access and quality, neighbourhood and built environment, healthcare access and quality, and structural discrimination and racism. The social and community context encompasses the food environment, social environment and cohesion, transportation instability, financial strain, food insecurity, and housing instability. Lived personal experience refers to everyday discrimination and stigma, neighbourhood perception, health literacy, implicit bias, social needs, and perceived health status. Together these factors contribute to the biology of adversity, healthcare access, health behaviours, and CV risk.

The risk of progressive CKD and KF is also linked with SDH, with groups of lower socioeconomic status carrying the heaviest load (66). For example, socioeconomic status impacts on diet quality through the unavailability of nutritious foods in disadvantaged neighbourhoods (122). In addition, many countries still lack access to basic diagnostics, a trained nephrology workforce, universal access to primary healthcare, and KRT (66). To identify patients with social needs and link them to available resources, the AHA recommends systematic screening for SDH—including housing insecurity, food insecurity, education, financial resource strain, alcohol use, race, ethnicity, tobacco use and exposure, depression, exposure to violence, physical inactivity, social isolation, and stress (13).

### Early detection and risk factor control

Given the strong evidence of CKD and albuminuria being independent risk factors for CVD, the AHA and the European Society of Cardiology recommend measurement of eGFR and albuminuria to identify at-risk patients who would benefit from evidence-based therapies to lower CVD and CKD risks (123). Concerningly, assessment of albuminuria is inadequate in real-world practice (<10%), even in those with CKD, DM or hypertension (124,125). Awareness of CKD is also low, ranging from 7–20% across global populations (7). Without stronger efforts to detect kidney disease among people at risk, we will miss important opportunities to initiate and maintain therapy and save kidneys, hearts and lives (7,126).

While the AHA advises screening for CKM risk factors across the life course to promote prevention and management (13), studies have shown that both screening and treatment of risk factors are dismally inadequate. An analysis of hypertension care in 44 LMICs found that among those with hypertension, 74% had ever had their blood pressure measured, 39% had been diagnosed with hypertension, 29% had received treatment, and 10% had controlled hypertension (127). In general, Latin American and Caribbean countries achieved the best performance relative to their predicted performance based on GDP per capita, whereas countries in sub-Saharan Africa performed the worst (127). The problem extends to HICs, as demonstrated by an analysis of 12 such nations, which found that even in the best-performing countries, treatment coverage was at most 80% and control rates were less than 70% (128).

Similarly with DM, around 6 in 10 of those with the condition are actually being diagnosed (129). In HICs, recommended targets for risk factors such as glycaemic control and blood pressure control are achieved by 50–70% of patients, with only ~20% meeting all recommended targets. LMICs fare worse, with just half of patients having good glycaemic control and one in four having reasonable blood pressure control (129). Only 12% of people living with diabetes were using statins.

**Figure 6:**
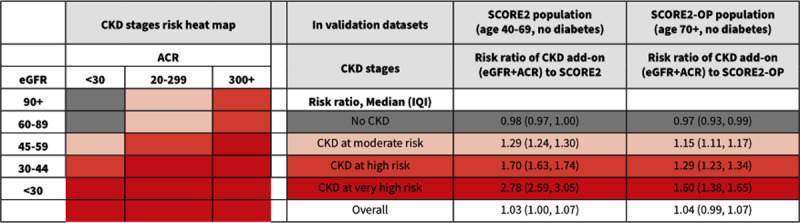
Cardiovascular risk stratified by presence or absence of kidney disease and/or albuminuria (42). Adapted from the European Journal of Preventive Cardiology, volume 30, issue 1, author: Matsushita K et al., Title: Including measures of chronic kidney disease to improve cardiovascular risk prediction by SCORE2 and SCORE2-OP, pages 8–16, Copyright 2022, with permission from Oxford University Press.

Novel calculators to predict CV risk have been developed that incorporate CKD measures. Adding eGFR or eGFR and UACR improved the predictive ability of both the systemic coronary risk estimation 2 (SCORE2) and SCORE2 in older persons (SCORE2-OP) algorithms (Figure 6) (42). It is important to note that the SCORE2 predictive tool performs poorly in poor socioeconomic subgroups and certain ethnic minorities (130). This underscores the importance of validating CV risk predictive tools in low-income countries and LMICs.

The AHAs new CVD risk calculator, Predicting Risk of Cardiovascular Disease Events (PREVENT) encompasses kidney and metabolic diseases and is based on data from a larger and more diverse sample than prior calculators (131). The PREVENT equations permit estimation of 10- and 30-year risk for total CVD (combined atherosclerotic CVD and heart failure) among adults 30 to 79 years of age, and include eGFR as a risk predictor, adjusting also for competing risk of non-CVD death (132). Importantly, where indicated, predictive utility is enhanced in additional models by inclusion of CKM indicators (urine albumin-to-creatinine ratio and hemoglobin A1c) or social determinants of health (as reflected by the social deprivation index). This model therefore may be capturing risk more holistically.

A major barrier to implementation of risk scores in many low resource settings is access to primary care facilities and to basic diagnostic tests, especially if out of pocket payment is required. Diagnosis is a critical step prior to implementation of therapeutic strategies; therefore, primary care must be strengthened and the required essential diagnostics and essential medications, most of which are common for CVD and CKD, must be made available under universal health coverage to ensure early and equitable access to quality care.

The authors of an African position paper on CKD highlighted that while assessment of UACR is recommended by many guidelines (133), it is unaffordable in African countries, and thus the use of urine test strips to identify CKD is considered acceptable (as it is in guidelines) and encouraged (134). Other authors have reported that in sub-Saharan Africa, CKD is often diagnosed very late, with kidney failure, due to resource constraints and nonavailability or unaffordability of primary care services to permit appropriate earlier identification (135). Many patients present late to nephrology services and die within weeks to months of their diagnosis, in part because of the absence of awareness, the lack of early detection among people at risk, lack of human resources and inadequate access to treatment for those with identified disease (136).

## Management of Kidney and Cardiovascular Diseases

**Figure 7:**
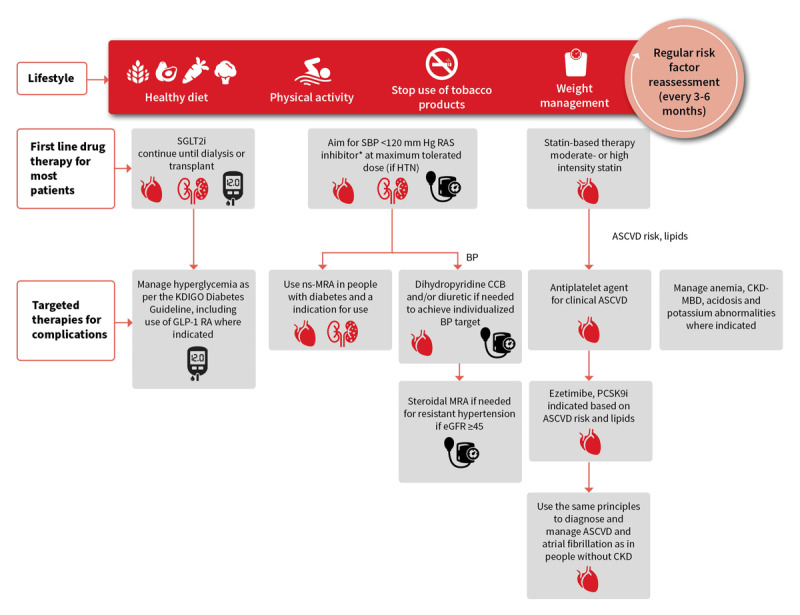
Holistic approach to CKD and CVD treatment and risk modification (137). ASCVD: atherosclerotic cardiovascular disease; BP: blood pressure; CCB: calcium channel blocker; CKD: chronic kidney disease; CKD-MBD: chronic kidney disease–mineral and bone disorder; CVD: cardiovascular disease; eGFR: estimated glomerular filtration rate; GLP-1 RA: glucagon-like peptide 1 receptor agonist; HTN: hypertension; KDIGO: Kidney Disease Improving Global Outcomes; MRA: mineralocorticoid receptor antagonist; ns-MRA: non-steroidal mineralocorticoid receptor antagonist; PCSK9i: proprotein convertase subtilisin/kexin type 9 inhibitor; RAS: renin-angiotensin system; SBP: systolic blood pressure; SGLT2i: sodium-glucose cotransporter-2 inhibitor. Adapted from https://www.kidney-international.org/article/S0085-2538(23)00766-4/fulltext. Author: Kidney Disease: Improving Global Outcomes (KDIGO) CKD Work Group. Title: KDIGO 2024 Clinical Practice Guideline for the Evaluation and Management of Chronic Kidney Disease. This figure is licensed under the CC BY-NC-ND license http://creativecommons.org/licenses/by-nc-nd/4.0/

Management and prevention of CKD and CVD demand a combination of therapies, lifestyle behaviours, and risk monitoring (Figure 7).

### Lifestyle changes

Lifestyle modification and weight loss are recommended by the AHA to address excess or dysfunctional adiposity and facilitate regression along CKM stages, which it defines as stage 0, no CKM risk factors; stage 1, excess or dysfunctional adiposity; stage 2, metabolic risk factors (hypertriglyceridaemia, hypertension, DM, metabolic syndrome) or moderate- to high-risk CKD; stage 3, subclinical CVD in CKM syndrome or risk equivalents (high predicted CVD risk or very high-risk CKD); and stage 4, clinical CVD in CKM syndrome (13). The CV benefits of physical activity in CKD and dialysis patients have been suggested in observational studies (138,139). In addition, low physical activity has been significantly associated with a higher likelihood of multimorbidity (140). A meta-analysis including 104 studies and 2,755,719 participants found that higher intake of potassium and vegetables, lower intake of sodium, increased physical activity, moderate alcohol consumption, and avoidance of tobacco smoking were associated with a lower risk of incident CKD in the community (141). These findings generally align with lifestyle recommendations to prevent CVD (142). A high potassium diet is usually not recommended in advanced CKD however, therefore caution and individualisation of recommendations is advised.

### Health literacy

Education is fundamental to health literacy, which enables individuals to make healthy choices (143,144). Education should be tailored to context and a patient’s own health literacy. Poor health literacy is common in patients with CKD, especially among those with low socioeconomic status and non-White ethnicity, and has been associated with reduced kidney function, poorer blood pressure control, decreased self-management, more frequent hospitalisation, and increased mortality (144,145). The benefits of public and professional education on kidney health were demonstrated in the ESCORT study, conducted in Thailand, in which patients with CKD received more comprehensive educational activities than the control group and health workers received training on CKD, clinical practice guidelines, and optimal diets (146).

The benefits of education extend to CVD. A study pooling six community-based cohorts in the US including 40,998 participants found that lower educational attainment was associated with lifetime CVD risk across adulthood, and higher education translated to healthy longevity (147). The authors suggested that education policies could lead to long-term health benefits, with opportunities for education in early life potentially having lasting effects in middle age and older age (147). A cluster-randomised, controlled trial in rural districts in Bangladesh, Pakistan, and Sri Lanka showed that an intervention that included home visits by trained government community health workers for blood pressure monitoring and counselling led to a greater reduction in blood pressure than usual care among adults with hypertension (148).

### Therapeutic interventions

Renin-angiotensin system inhibitors (angiotensin-converting enzyme inhibitors [ACEIs] and angiotensin receptor blockers [ARBs]) reduce proteinuria and GFR decline (149) and have benefits in treating hypertension, improving diabetic nephropathy and other forms of CKD, preventing or ameliorating congestive HF, and optimising prognosis after AMI (150). These agents have been the mainstay of treatment for CKD (beyond therapy for a primary kidney disease) since the 1980s (137,151), but novel therapies are proving to be game changers as reflected in the latest KDIGO CKD guidelines (137).

Therapy with SGLT2 inhibitors, non-steroidal MRA and GLP-1a have been shown to improve cardiorenal outcomes in CKD patients with and without diabetes. A meta-analysis of 78,607 participants from 11 phase 3 placebo-controlled trials of SGLT2 inhibitors has shown a reduction in major adverse cardiac events (HR 0.91 [95% CI 0.87–0.96], p < 0.0001) in diabetes, HF and CKD patients, establishing a role of SGLT2 inhibitors in all three patient groups [I^2^ = 0%] (152). Another meta-analysis of 13 SGLT2 inhibitor trials with 90,409 participants has shown a reduction of CKD progression (relative risk [RR] 0.63, 95% CI 0.58–0.69), AKI ([RR] 0.77, 0.70–0.84) and CV death with HF hospitalisation ([RR] 0.77, 0.74–0.81) in both patients with and without diabetes (153). In a randomised trial of 3,353 CKD patients with diabetes, GLP-1a reduced kidney disease progression [hazard ratio 0.79 (95% CI, 0.66–0.94)], death from CV causes [HR 0.71 (95% CI, 0.56–0.89)] and all cause death (hazard ratio, 0.80; 95% CI, 0.67 to 0.95, p = 0.01) (154). Finerenone, a non-steroidal MRA, in 13,206 CKD patients with diabetes reduced kidney disease progression [HR, 0.77; 95% CI, 0.67–0.88; p = 0.0002] and CV outcomes [HR, 0.86; 95% CI, 0.78–0.95; p = 0.0018]. Hyperkalaemia led to more frequent treatment discontinuation in patients receiving finerenone (1.7%) than placebo (0.6%) (155). A meta-analysis including 3,460 patients with HF and CKD found that compared with ARBs, the angiotensin receptor/neprilysin inhibitor (ARNI) sacubitril/valsartan significantly increased eGFR and decreased blood pressure and N-terminal pro b-type natriuretic peptide (NT-proBNP), indicating that it might have CV and kidney benefits in patients with HF and CKD (156). In partnership with other international organisations, ISN is developing tools to provide the necessary therapy (e.g RAAS inhibition) and interdisciplinary care of kidney patients with heart disease across all six regions of the World Health Organization (157).

Additional new therapies have been developed for HfrEF (e.g. Omecamtiv-Mecarbil, Vericiguat, Ivabradine), which have improved mortality, CV death or hospitalisation for HF, however in general these trials have only included patients with up to CKD3B (158). Such frequent exclusion of patients with more advanced CKD leaves an important knowledge gap in the treatment of CVD in those at the highest risk and in greatest need of effective therapies.

A study by Patel et al. demonstrated that despite their elevated risks of mortality, patients with both HFrEF and kidney disease were suboptimally treated with evidence-based medical therapies, even at eGFR levels where such therapies would not be contraindicated (37). Given the very high CV burden and risk of death among patients living on dialysis or with kidney transplants, large scale trials are required to examine the safety and efficacy of these medications in these populations.

## Access and Affordability of Medicines

Global inequities in the availability of medications fuel disparities in disease prevalence and outcomes. It is estimated that nearly two billion people globally have no access to essential medicines (159). An African Association of Nephrology position paper from five African regions highlights that financial constraints limit CKD management strategies to lifestyle changes (weight control, dietary restrictions, exercise, and smoking cessation), maintaining target levels of blood pressure and glucose, avoiding exposure to nephrotoxins, and pharmacological intervention with ACEIs/ARBs (134). The experts cited limited insurance coverage, poor accessibility, and high out-of-pocket expenditure as barriers to adopting SGLT2 inhibitors.

Data from the global, observational DISCOVER study of patients with DM showed that countries in Africa and Asia had notably low rates of SGLT2 inhibitor and GLP-1 RA use, and there was a trend toward higher use in countries with greater economic resources (160). Access to these agents is particularly important in LIC and LMIC to prevent progression to KF since access to KRT is either rationed or simply unaffordable and not available. Importantly, Africa has been excluded from many studies (161).

A survey of 213 healthcare workers from 14 LICs and 25 LMICs showed that access to essential kidney medicines was challenging, especially in community settings, where only 31% of respondents reported access was reasonable (162). More than two-thirds of respondents reported barriers to access relating to national health policy and funding, regardless of healthcare setting, while in community settings, more than 70% cited barriers relating to resourcing and demand (162).

A polypill containing blood pressure-lowering medication, a statin, diuretic, and beta blocker has been discussed as a potential affordable strategy for CVD prevention in LMICs; however, issues that may limit its implementation worldwide include dosing of the individual components and patents for each drug (163).

## Implications for Patients

### Out-of-pocket costs

High out-of-pocket costs mean that some patients cannot afford evidence-based medicines for CKD and CVD. In an analysis of the Prospective Urban Rural Epidemiology (PURE) study conducted in 18 countries, cardioprotective medicines were potentially unaffordable for one-third of LMICs and 60% of households in LICs (164). A further analysis in 163,466 participants of the PURE study found that essential CVD medicines were unavailable and unaffordable for a large proportion of communities where individuals at high CVD risk reside, particularly in LMIC and LIC (165). After accounting for sociodemographic and economic factors, education and comorbidities, the unavailability, and unaffordability of essential CVD medicines were associated with a higher risk of major adverse CV events (165). Husain and colleagues reported that the costs of one month’s antihypertensive medications were, on average, 6.0 days’ wages for brand medicines and 1.8 days’ wages for generics (166). Affordability was lower in LICs and LMICs than HICs and UMICs for both brand and generic medications (166).

Across Africa, there is significant variation in the cost of SGLT2 inhibitors, with generics imported from India and Pakistan being more affordable (135). In many cases, prices are higher than the median daily income.

Patients in HICs are not immune to the impact of medicine pricing (7). In the US, a one-month supply of an SGLT2 inhibitor costs USD 530–650, and for finerenone, USD 580–690 per month, while GLP-1 RAs cost USD 770–1300 per month, leading to insurers’ reluctance to pay (6). In addition, monthly co-payments can amount to hundreds of dollars per month for each medication, making the use of multiple drugs a massive challenge. Note that prices vary across different HICs, depending on the socio-political environment.

An analysis of 66 studies examining the effect of NCDs on household economics in LMICs reported that >60% of patient populations with NCDs experience catastrophic health expenditure (167). Such expenditure was greater in lower- compared with higher- income groups. The highest incidences of catastrophic health expenditure were found in low-income patients with CVD in Tanzania (92%), India (92%), and China (79%). A study focused on sub-Saharan Africa and defining catastrophic health expenditure as health expenditure above 5%, 10%, and 25% of total household expenditure, found that households with CVD were three times more likely to incur catastrophic health expenditure compared with households without CVD (168). When categorised by the extent of catastrophic health expenditure, households with CVD had two- and three-fold odds of incurring catastrophic health expenditure at the 5% and 10% thresholds, respectively, and four-fold odds at the 25% and 40% thresholds (168). It is estimated that ~188 million people in LMICs experience catastrophic health expenditure every year due to CKD (162).

Out-of-pocket costs are a barrier to treatment and a driver of non-adherence and discontinuation, with poorer households affected most (169). An analysis of 14 studies from HICs and LMICs found that cost adversely influenced adherence to CKD medication and dialysis treatment (169). In poorer countries, patients who did receive treatment were typically diagnosed late, under-dialysed and had high levels of mortality (169). A systematic review of how patients in LMICs deal with the healthcare costs of chronic illness reported that in addition to selling possessions and borrowing from friends and family, coping strategies included taking children out of school, stopping or delaying treatment, and seeking alternative treatment for conditions like hypertension (170). Not surprisingly, multimorbidity increases out of pocket costs and risks for catastrophic expenditure, highlighting the need for integrated care, appropriate guidelines to support rational use of medication, and most importantly universal health coverage to ensure affordable and continued access to the life-saving medications required for CVD and CKD (171,172).

### Indirect costs—unemployment

CKD and CVD also come with substantial indirect costs, including the loss of employment, mental strain, and stress. A review of 260 CKD studies from 30 countries reported that in some patients, treatment led to unemployment, and patients feared catastrophic events because of limited finances (173). Those able to continue working often had informal or temporary jobs with reduced income, while others were forced into unemployment (173). Pain, fatigue, anxiety, and depression limited the ability of patients with KF to conduct daily activities, including their paid job (173).

A study of 36,732 adults aged ≥30 years who participated in the Korean National Health and Nutrition Examination Survey between 2014 and 2021 found that patients with CKD had a two-fold odds of unemployment compared to those without CKD (174). A study in nine nephrology clinics in the Netherlands among working age (18–67 years) patients with CKD Stage G3b-G5, dialysis or transplant reported that two in three (65%) were employed but reported moderate work ability (175). Of those, 21% received disability benefits, 37% were severely fatigued, 7% expected to drop out of the workforce, and 49% experienced CKD-related work limitations. Adjustments at work included fewer hours, working at a slower pace, changes to work tasks or schedules, and working from home.

Studies have reported employment rates of between 19% and 33% among patients on dialysis (176,177,178,179). A study from India reported 60% and 64% employment rates in haemodialysis and peritoneal dialysis patients, respectively, before the initiation of treatment (179). After dialysis commenced, loss of employment was observed in 44% of haemodialysis and 51% of peritoneal dialysis patients (179). A study of 480,597 patients from the US Renal Data System (USRDS) registry initiating dialysis at various time points between 1996 and 2013 found that on average over the entire study period, 38% of patients employed six months before KF stopped working before starting dialysis (180).

Numerous studies have indicated that CVD has an impact on patients’ ability to return to work (181). Among 11,880 patients with a first-time HF hospitalisation, 68% returned to work within one year and 25% did not (182). Patients with longer/higher education were twice as likely to return to employment as those with basic school education, while those with CKD were half has likely to go back to work (182). It has also been shown that an initial return to work may be followed by leaving employment. A nationwide Danish registry study found that 91% of patients with a first-time AMI returned to work within one year, but one year after their return, 24% were detached from employment and receiving social benefits (183). High education level and high income favoured continued employment (183). Similarly, among 36,174, 30-day survivors of first-time hospitalisation for AF, 34,924 (96.5%) patients returned to work (184). Of those who returned to work, 23,905 (68.5%) left the workforce within a median of 1.5 years (184). Higher education levels were associated with a lower risk of detachment from the workforce relative to lower education levels (184).

A review of vocational reintegration of patients experiencing an ACS noted that cardiac events raised the likelihood of poorer professional conditions including reduced responsible area, part-time employment, lower salary and discharge from jobs (181). An analysis of 497 patients with acute or chronic ischaemic heart disease estimated that the mean work productivity loss was €9,673 per person (185).

### Indirect costs—psychosocial aspects

Psychosocial stress—including symptoms of mental disorders and stressors such as loneliness and critical life events—is associated, in a dose-response pattern, with the development and progression of CVD, independently of conventional risk factors and sex (142). An analysis of 2,585 patients with CKD stages 2–4 but without CVD enrolled in the Chronic Renal Insufficiency Cohort (CRIC) found that one in four had depression at baseline (186). Those with depression were more likely to be women (56% vs. 46%), non-White (68% vs. 53%), with household income <USD 20,000 (53% vs. 26%), without a high school degree (31% vs. 15%), uninsured (13% vs. 7%), with lower eGFR (42 vs. 46 mL/min/1.73 m^2^), and with higher UACR (90 vs. 33 mg/g) compared to patients without depression. In multivariate analyses, depression was associated with a 29% increased risk of developing CVD (186). Data from Finland, France, Sweden, and the UK have shown that work stress, which refers to job strain or effort-reward imbalance at work, indicated that in men with cardiometabolic disease, age-standardised mortality rates were substantially higher in those with job strain (149.8 per 10,000 person-years) compared to those without (97.7 per 10,000 person-years; mortality difference 52.1 per 10,000 person-years; multivariable-adjusted HR 1.68, 95%CI 1.19, 2.35) (187).

## Global Implications of Health Policy for Kidney and Cardiovascular Health

The rising prevalence of both CVD and CKD places an enormous burden on healthcare systems (1,2). In addition, the impact on the social and financial systems within countries is similarly impacted though difficult to quantify. The UNs 17 SDGs outline an overarching aim of fostering healthy populations living in a sustainable planet (118). Target 3.4 is to reduce premature mortality from NCDs by one-third by 2030, while target 3.8 is to achieve universal health coverage, including financial risk protection, access to essential healthcare services and access to safe, effective, quality and affordable essential medicines and vaccines for all (118). LICs and LMICs bear a disproportionate burden of CKD and CVD, and thus, the achievement of these goals is an enormous challenge (2,188,189).

The WHO’s Global Action Plan for the Prevention and Control of NCDs 2013–2020 was adopted by the World Health Assembly in 2013 (190). The plan outlines six objectives, of which the fourth is ‘to strengthen and orient health systems to address the prevention and control of noncommunicable diseases and the underlying social determinants through people-centred primary healthcare and universal health coverage’ (190). Nine global targets to be achieved by 2025 are highlighted (190). These include a 25% relative reduction in the risk of premature mortality from NCDs, at least 50% of eligible people receiving drug therapy (e.g. glycaemic control) to prevent heart attacks and strokes, 80% availability of affordable basic technologies and essential medicines, halting rises in diabetes and obesity, and targets related to risk factors such as tobacco, physical inactivity, salt, alcohol, and raised blood pressure. The plan provides a road map and policy options intended to assist member states attain these targets and to realise SDG 3—Good Health and Wellbeing (190,191). Kidney disease has not been included in these priority conditions, which has hindered progress in integrating the detection and treatment of CKD into primary care (119).

The recommended interventions in the Global Action Plan were updated and endorsed in 2017 as the WHO ‘Best Buys’ (191). The overarching actions to enable implementation of objective four encompass health financing mechanisms, early detection and behavioural risk factors, health system capacity, availability of essential medicines, and the use of digital technologies (191). As an example, drug therapy to control blood glucose levels and hypertension is recommended as a cost-effective intervention for individuals who have had a heart attack or stroke or are at high risk (≥30%) of a fatal or non-fatal CV event in the next 10 years (191).

The WHO ‘Saving lives, spending less’ document, published in 2021, detailed the health and economic benefits of investing in the ‘Best Buys’ in LMICs (192). According to the document, an additional investment of USD 0.84 per person per year in LICs and LMICs could deliver a package of measures to reduce the burden of NCDs (192). Approximately 5% of annual domestic government health expenditure would be required in these countries (192). In LICs alone, the average per capita investment would be even less, at USD 0.51 annually, while in LMICs, the per person average would be USD 0.90. Three-quarters of overall health spending in LICs and 97% in LMICs is covered by domestic financing, of which out-of-pocket payments form the largest component in many countries, particular LICs and LMICs (193). The WHO Global Coordination Mechanism on NCDs financing working group has argued that insurance and government provision should replace out-of-pocket expenditure as the main funding source in LMICs, meaning that governments must raise more revenue for NCDs.

An analysis in the 194 WHO member states from 2015 to 2020 reported that implementation of NCD policies increased from 39.0% in 2015 to 45.9% in 2017 and 47.0% in 2020 (194). Implementation was lowest for lifestyle-related policies (e.g. tobacco, unhealthy foods, and alcohol) and progress had reversed for a third of all policies. The mean implementation score rose with each successive World Bank income group. Implementation tended to be highest in HICs with lower premature NCD mortality, and in democratic states with well-resourced and managed health systems. Low-income and less democratic countries had the lowest policy implementation. Overall, less than a third of WHO-backed NCD policies had been fully implemented in 2020.

### Cost and cost-effectiveness for health systems

A study of patient payments for services in the public and private sectors in Kenya found that NCD screening costs ranged from USD 4 to 36 (195). Annual hypertension medication costs ranged from USD 26 to 234 and USD 418 to 987 in public and private facilities, respectively (195). Stroke admissions (USD 1,874 vs. 16,711) and dialysis for CKD (USD 5,338 vs. 11,024) were among the most expensive treatments (195). The study reported that a large proportion of Kenyans aged 15 to 49 years do not have health insurance, making NCD services unaffordable for many considering the high cost of services relative to income (in 2013, the estimated average household expenditure per adult was USD 413; USD 721 in urban areas and USD 272 in rural settings) (195). Case-finding and early treatment of CKD in high-risk populations has been found to be cost effective in some high and upper middle-income countries. This has not been studied in lower resource settings but should be presumed to be cost-effective until proved otherwise (196,197,198).

A review of the literature published in 2020 indicated that approaches to control hypertension can be a cost-effective way to prevent premature CVD in LMICs across a variety of population, clinical, and health system contexts (199). Most interventions that reported cost per averted disability-adjusted life-year (DALY) were cost-effective using national income thresholds, with costs per averted DALY not exceeding the average GDP per capita of LMICs (199). A cost analysis of the WHO HEARTS programme for hypertension control and CVD prevention in Ethiopia reported that the estimated annual cost per adult primary care user was USD 5.3 for hypertension control and USD 19.3 for integrated CVD risk management (200). The estimated cost of medication per person treated for hypertension was USD 9.0, whereas treating DM and high cholesterol would cost USD 15.4 and USD 15.3 per person, respectively. More than one-third (37%) of the total cost of the hypertension control programme was attributable to medications.

In the US and UK settings, the risk of CV morbidity and the costs and bed days associated with its management increased substantially with CKD severity (201). Additional costs and bed days were largely driven by high levels of albuminuria and increased severity of CKD (201). For example, patients with stage 5 CKD and UACR values >300 required an estimated additional 803 and 1,017 bed days per 1,000 patient-years for the management of CV morbidity in the US and UK, respectively, versus stage 1 (or without) CKD, and normoalbuminuria (201).

A secondary analysis of 28,261 years of data from the Study of Heart and Renal Protection (SHARP) randomised trial, conducted in 7,246 patients from Europe, North America, and Australasia, reported that annual hospital costs in CKD patients without DM or vascular disease ranged from £403 in CKD stages 1–3b to £525 in CKD stage 5 (not on dialysis) (202). Non-fatal major vascular events increased annual costs in the year of the event by £6,133 for patients on dialysis and by £4,350 for those not on dialysis and incurred additional costs in subsequent years (202).

### [BOX] Case study: UK statistics on heart and kidney diseases

In 2024, 7.6 million people have heart and circulatory diseases in the UK which is twice as many as cancer and Alzheimer’s combined; and half of the population develop a heart or circulatory condition during their lifetime. Heart and circulatory diseases cause 27% of all deaths in the UK; 170,000 deaths every year, 480 every day, and 1 every 3 minutes. Heart failure is a major concern affecting over one million people. Heart and circulatory diseases cost £10 billion each year for healthcare and total cost to the economy is £25 billion/year (203).

In 2023, 7.2 million people are living with kidney disease in the UK; 30,000 adults and children are on dialysis. Dialysis costs £34,000 per year. The number of people requiring dialysis could rise to 143,000, and the number requiring transplantation could be as high as 12,000/year by 2033. The cost of kidney disease in the UK is £7.0 billion, with £6.4 billion being direct costs to the NHS—about 3.2% of the NHS budget. In addition, missed work by patients with CKD and their care givers contributes to an annual productivity loss of £372 million. (204)

## Potential Solutions

### Recognize kidney disease as an important contributor to the global burden of disease

As discussed above, progress has been made in improving outcomes for people living with CVDs, but the age-standardised prevalence and death rates for CKD and DM continue to rise. Similarly, data from the ARIC study, heavily quoted here, and a more recent meta-analysis (14), highlight the close interconnection between heart and kidney diseases that has been known for two decades but has only recently become part of the more mainstream discourse. A major contributor to this fact is that people with kidney disease have been historically excluded, or significantly underrepresented, in CVD studies (205). This needs to change. Many barriers exist to achieving the goal of early detection and timely treatment for all with kidney and CV diseases. Strategies to close these gaps are highlighted below (Table 2).

**Table 2:** Policy solutions.


POLICY LEVEL		

**Global/National Policy**	Achieve SDGs and tackle climate change	Implement policies promoting healthy environments (e.g., air quality, active transport, green spaces) Foster sustainable food systems to reduce diet-related NCDs

	Address social determinants of health	Reduce poverty and health inequities through targeted social policies Invest in education and eliminate discrimination to improve health literacy and access

	Understand disease burden	Recognize CKD as a contributor to the global disease burden and as an important modulator of CVD risk

	Public health strategies	Promote healthy diets (e.g., regulate sodium, sugar, and trans fats) Enforce tobacco control Design safe urban spaces for physical activity Implement large-scale obesity prevention and public education campaigns

	NCD policies and planning	Integrate CKD and CVD prevention and management into national NCD strategies Ensure dedicated funding and resources for CKD and CVD within NCD budgets and programmes

**Health systems level**	Track disease burden and costs	Develop and maintain CKD and CVD registries to track incidence, outcomes, and disparities Conduct economic evaluations to assess the cost-effectiveness of integrated care and interventions

	Strengthen Integrated care	Incorporate CKD into existing CVD and broader NCD programmes and care pathways Facilitate multidisciplinary, person-centred care models that address comorbidities

	Support quality clinical care	Provide continuous education and training for the health workforce on CKD-CVD interactions Integrate clinical guidelines to support simplified, coordinated, and holistic care delivery Allocate adequate resources for screening, diagnosis, and long-term management

	Enable effective workforce strategies	Promote task sharing and task strengthening to optimise the use of non-physician providers Expand the role of primary care and community health workers in early detection and management


#### Standard of care and guidelines

Nephrologists have long been aware of the substantial risk of CVD with CKD, and management of CVD risk has been included in CKD guidelines for many years. The management of CKM should involve collaboration across clinical guidelines and medical specialties. Traditionally, management of these patients has been in silos with separate guidelines focusing on heart disease, diabetes, hypertension and CKD, and the same patient having to visit multiple clinicians to receive the necessary fragmented care. Such processes have not fostered holistic care or efficiency and have overburdened the primary care work force (126). Newer guidelines for CKD and CVD and the Global Diabetes Compact include more holistic approaches to patient management, incorporating the diverse risks associated with the CKM conditions (137,142,151,206,207,208). Simplified or integrative guidelines should permit streamlining of care for CVD, CKD and their risk factors at primary care level which should improve efficiency, reduce clinical burden and improve patient experience and outcomes (126).

#### Risk stratification and outcome monitoring

Management should be patient centred. CVD and CKD risk should be considered within the context of a holistic assessment of the SDH and the life course approach. All patients with one of the three conditions (cardiac disease, kidney disease or metabolic syndrome) should be screened for the others. Blood pressure and body weight must be regularly monitored and optimally controlled in all individuals. For all patients, eGFR, albuminuria or urine dipsticks should be determined regularly. In those with HF, evaluating volume management, CV health, and HF status impacts prognosis. People with DM must be monitored for optimal glucose management as well as screened regularly for additional complications include eye checks, foot care and infection, and GFR and UACR. The burden of SDH should be considered in each individual, using concepts such as the ‘polysocial’ or the ‘Whole Person’ risk score, and barriers to optimising health and healthcare should be addressed (209,210).

#### Patient-centred interventions

Effective interventions should be tailored to individual patients, taking into consideration their life stage, personal values, social context, and level of health literacy. It is reasonable to suggest that close monitoring of outcomes and implementation of patient-centred strategies will improve care for those with the triad of CV, kidney and metabolic diseases. Improved understanding of the ‘CKM’ syndrome among all clinicians including internists, primary care physicians, cardiologists, nephrologists, diabetologists, specialist nurses, and other allied healthcare professionals will help with risk factor evaluation, management, and initiation of appropriate drug therapy. Every encounter with clinicians for the patients with CKM syndrome should be an opportunity to identify interventions, which will help to mitigate the consequences in a swift and ideally equitable fashion. Examples of actions to improve health literacy and patient empowerment are outlined in Table 3.

**Table 3:** Informing and empowering patients.


FACTORS THAT SHOULD BE ADDRESSED TO EMPOWER PATIENTS AND CARE GIVERS	RECOMMENDATIONS

**Peer mentoring**	Establish support groups specifically tailored for individuals living with both CKD and CVD, as well as their care partners. These groups can provide a platform for sharing experiences, coping strategies, and emotional support.

**Patient-centred research initiatives**	Involve patients and care partners in the design and implementation of research initiatives focused on CKD and CVD. Their insights can offer valuable perspectives and ensure that research efforts address their specific needs and concerns.

**Education and information resources**	Develop educational materials and resources that provide comprehensive information about managing both CKD and CVD. These resources should be easily accessible and available in multiple formats to accommodate different learning preferences.

**Advocacy and policy engagement**	Empower patients and care partners to advocate for policies and initiatives that improve access to quality care, treatment options, and support services for individuals living with both CKD and CVD.

**Care coordination and communication**	Enhance communication and collaboration between healthcare providers, patients, and care partners to ensure continuity of care and a holistic approach to managing both conditions. Encourage open dialogue and shared decision-making processes.

**Addressing social and financial barriers**	Recognise and address the social determinants of health and financial challenges that may impact individuals living with both CKD and CVD and their families and/or caregivers. Provide resources and support to help navigate these barriers effectively.

**Promote self-management and empowerment**	Offer self-management programmes and resources that empower patients and care partners to take an active role in managing their health and wellbeing. This can include lifestyle modification strategies, medication adherence support, and self-monitoring tools.

**Cultural competency and diversity**	Ensure that support services and resources are culturally competent and inclusive of diverse perspectives and backgrounds. This can help foster a sense of belonging and improve the overall experience for patients and care partners.

**Regular feedback and evaluation**	Establish mechanisms for collecting feedback from patients and care partners about their experiences with healthcare services and support programmes. Use this feedback to continuously improve and tailor interventions to meet their evolving needs.

**Holistic wellness approach**	Recognise the interconnectedness of physical, emotional, and social wellbeing in individuals living with both CKD and CVD. Adopt a holistic approach to care that addresses all aspects of health and promotes overall wellness.


#### Health systems strengthening

Integration of kidney and CV care within existing health systems programmes such as those directed at hypertension, DM, HIV, and tuberculosis would create opportunities to enhance quality of care, strengthen capacity of all levels of healthcare workers, and improve affordability, acceptability, and adherence from the patient perspective. Improving health for women and children is also required to reduce the CKM burden worldwide. Health systems’ resilience must be fostered to protect populations from outbreaks and humanitarian emergencies which impact everyone living with chronic diseases.

#### Population health implications

Considering the broader picture of cardiac and kidney risk reduction is essential. These conditions share common risk factors of high blood pressure, obesity, life course determinants, and the SDH. Public health strategies are required to modify unhealthful food content and incentivise and facilitate healthy lifestyle practices. Global collaborative strategies are needed to address the social determinants of health and tackle climate change which have far reaching impacts for people at risk of or living with heart and kidney diseases (Figure 3).

#### Considerations to enhance global equity and solidarity

Professional societies and global health organisations must raise one voice to improve access to early diagnosis and treatment for kidney and CV diseases. Prices for life-saving medications must be fair and these medications should be accessible to all without experiencing financial hardship. Addressing poverty, nutrition and equity, and achieving all other SDGs are imperative to develop comprehensive, equitable and long-term strategies to promote wellbeing and reduce the burdens of kidney and heart diseases now and for future generations.

## Conclusion

Heart disease and kidney disease are closely interlinked—they share many common risk factors and many common therapeutic approaches, and each modifies the risks and outcomes of the other. Both represent a spectrum of common diseases, impacting millions of people globally. Both diseases are preventable if shared risk factors are diagnosed and treated early. As such, a holistic approach to detecting and managing common risk factors for kidney and heart disease such as diabetes, hypertension and obesity is key at both the public health and the health systems levels. Access to diagnosis and quality care for both is inequitable across the globe. Early diagnosis of kidney and heart disease is increasingly important as newer medications are game-changers, improving short- and long-term outcomes, but these remain out of reach for many because of unaffordability. Patients and the public must be educated and empowered to protect their hearts and kidneys. Cardiovascular and kidney care integration can yield many benefits in terms of saving lives and money. Now is the time to unite and support heart and kidney health globally.
